# Walking with Different Insoles Changes Lower-Limb Biomechanics Globally in Patients with Medial Knee Osteoarthritis

**DOI:** 10.3390/jcm12052016

**Published:** 2023-03-03

**Authors:** Guillaume Jaques, Baptiste Ulrich, Laurent Hoffmann, Brigitte M. Jolles, Julien Favre

**Affiliations:** 1 Swiss BioMotion Lab, Lausanne University Hospital and University of Lausanne (CHUV-UNIL), CH-1011 Lausanne, Switzerland; 2 NUMO Systems, CH-8953 Dietikon, Switzerland; 3 Institute of Electrical and Micro Engineering, Ecole Polytechnique Fédérale Lausanne (EPFL), CH-1015 Lausanne, Switzerland; 4 The Sense Innovation and Research Center, CH-1007 Lausanne and CH-1950 Sion, Switzerland

**Keywords:** adduction moment, ankle, eversion moment, flexion angle, flexion moment, footwear, hip, knee, osteoarthritis, lateral wedge, personalized intervention, walking

## Abstract

Using insoles to modify walking biomechanics is of keen interest for the treatment of medial-compartment knee osteoarthritis. So far, insole interventions have focused on reducing the peak of the knee adduction moment (pKAM) and have led to inconsistent clinical outcomes. This study aimed to evaluate the changes in other gait variables related to knee osteoarthritis when patients walk with different insoles to provide insights into the necessity to enlarge the biomechanical analyses to other variables. Walking trials were recorded for 10 patients in four insole conditions. Changes among conditions were computed for six gait variables, including the pKAM. The associations between the changes in pKAM and the changes in the other variables were also assessed individually. Walking with different insoles had noticeable effects on the six gait variables, with high heterogeneity among patients. For all variables, at least 36.67% of the changes were of medium-to-large effect size. The associations with the changes in pKAM varied among variables and patients. In conclusion, this study showed that varying the insole could globally influence ambulatory biomechanics and that limiting measurement to the pKAM could lead to an important loss of information. Beyond the consideration of additional gait variables, this study also encourages personalized interventions to address inter-patient variability.

## 1. Introduction

Knee osteoarthritis (OA) is a prevalent disease causing pain and disability and reducing the quality of life of millions of individuals worldwide [1]. While its etiology is not fully understood, ambulatory loading has been shown to be an important factor in disease progression, and modifying walking biomechanics constitutes a concrete treatment option for this currently incurable disease [2,3].

The peak knee adduction moment (pKAM) during the first half of the stance phase is the gait variable that has received the highest attention in the treatment of medial-compartment knee OA, which is the most frequent form of the disease [4]. Indeed, the pKAM has been related to the severity, progression, and pain of medial knee OA [5,6,7], and various therapeutic interventions have been introduced to reduce this variable [8]. Among them, footwear interventions, particularly insoles, are very attractive, because they can modify walking patterns during daily activities without hindering comfort, therefore facilitating high compliance. Unfortunately, even though insoles have been repetatedly shown to reduce the pKAM in groups of patients with medial knee OA [9,10,11], inconsistent clinical results have been reported [12,13]. Therefore, in view of the empirical advantages that insoles provide to modify ambulatory biomechanics in knee OA patients, there is a need to better understand this intervention in order to enhance its clinical outcomes, either by improving insole designs or by identifying patients more likely to benefit from an insole intervention.

With prior studies focusing on the pKAM, little is known about the effects of insoles on the other gait variables, particularly those that have been associated with medial knee OA progression; namely, the impulse of the knee adduction moment during stance phase (iKAM), the peak knee flexion moment during the first half of stance phase (pKFM), and the peak knee extension angle around heel-strike (pKEA) [5,14,15]. So far, the iKAM has been reported to decrease with lateral wedges in multiple studies [9,11,16]. Additionally, a study on lateral wedges reported a correlation between changes in pKAM and iKAM, with larger reductions in iKAM observed in patients with larger pKAM reductions [17]. Beyond that, there is a paucity of data on the pKFM and pKEA, as well as on other types of insoles [18]. Furthermore, although there could be significant differences among patients in their responses to insole interventions [17,19], to the authors’ knowledge, no study has individually analyzed the patients. Therefore, it remains unknown if excluding the other variables associated with medial knee OA is appropriate, because it simplifies the analyses, or if it is a mistake, because important biomechanical information is lost. For example, if an insole decreases the pKAM but at the same time increases the iKAM, pKFM, and/or pKEA, the benefits could be thwarted [20,21]. Consequently, as a first step toward determining the need for a more global consideration of knee biomechanics, there is a need to more comprehensively characterize the changes in the other gait variables. If changes occur, then it will also be necessary to assess whether the changes in these additional variables are independent of the changes in pKAM.

Gait interventions for knee OA can change ankle and hip kinetics in a way that can contribute to faster progression of existing OA or to the onset of OA at these adjacent joints [22]. Therefore, there is an interest in extending the gait variables analyzed in this study to the peak ankle eversion moment (pAEM) and peak hip adduction moment (pHAM) during the first half of stance phase [23]. So far, changes in these variables have mainly been characterized for lateral wedges, with reports of decreases in pAEM and inconsistent changes in pHAM [11]. Additionally, one study reported an absence of inter-patient correlation between changes in pAEM and pKAM with lateral wedges [24]. Thus, there remains a need to increase our understanding of pAEM and pHAM changes by testing diverse types of insoles and describing intra-patient relationships.

The primary objective of this study was to provide insights into the necessity to examine the iKAM, pKFM, pKEA, pAEM, and pHAM in addition to the pKAM when modifying the walking biomechanics of medial knee OA patients with insoles. Specifically, this study aimed at evaluating the individual changes in iKAM, pKFM, pKEA, pAEM, and pHAM when patients walk with different insoles and describing the relationships between these changes and changes in pKAM. A secondary objective was to further our understanding of lateral wedge insoles, the most prominent type of insoles in the medial knee OA literature, by assessing the inter-patient relationships between changes in pKAM and changes in the other gait variables, particularly pKFM and hKFA.

## 2. Materials and Methods

### 2.1. Patients

Ten patients with unilateral or bilateral primary medial knee OA of Kellgren–Lawrence grades I to III [25] were continuously recruited for this IRB-approved study. Individuals with a history of lower limb surgery, neurological disorders, or use of walking aids were excluded. An index knee was defined for each patient based on higher OA severity or stronger pain in case of equal disease severity. The characteristics of the participants and index knees analyzed in this study are provided in Table 1.

### 2.2. Gait Analysis

Walking biomechanics was analyzed for the lower extremity of the index knee in a gait lab including a 10 m long walkway instrumented with a 14-camera motion capture system (Vicon, Oxford, UK) and two floor-embedded forceplates (Kistler, Winterthur, Switzerland) synchronously recording at 120 Hz and 1200 Hz, respectively.

Patients were asked to walk through the walkway in their personal shoes with four different insole conditions. The first condition (a) was the shoes (sneaker type) worn by the patients on the day of the test. For the three other conditions, an element was inserted bilaterally between the midsole and the comfort insole of the patients’ shoes. The added elements were (b) a full-length arch support insole (AFT International, Ranst, Belgium), (c) the arch support insole on top of a full-length 5° lateral wedge insole (NUMO Systems, Dietikon, Switzerland), and (d) a custom-made insole for medial knee OA intended to realign lower extremities (NUMO Systems, Dietikon, Switzerland).

Trials at self-selected normal, slower-than-normal, and faster-than-normal walking speeds were collected for the four insole conditions in randomized order. For each walking speed, three successful trials were recorded, leading to a total of 36 trials collected per patient. A trial was considered successful if the foot of interest fully stepped on a forceplate.

Before recording the trials, patients were equipped with clusters of reflective markers following a standard protocol [26]. The clusters served to measure the movement (position and orientation) of technical frames embedded in the thigh, shank, and foot segments. The anatomical frames of the lower-extremity segments, as well as the technical-to-anatomical transformations, were defined during a standing reference pose using additional markers placed on anatomical landmarks [26]. During the trials, the movements of the anatomical frames were computed using the movement of the technical frames and the technical-to-anatomical transformations [27,28]. The knee flexion angle and the joint moments were determined following standard calculations based on the anatomical frame movements, forceplate data, and inertia properties of the segments [29,30]. Moments were expressed in the anatomical frame of the distal segment and normalized to bodyweight and height (%BW⁄Ht). The pKAM, iKAM, pKFM, pHAM, pAEM, and pKEA gait variables were extracted for each trial during the stance phase on a forceplate. Biomechanical processing was conducted with the software application BioMove (Stanford, CA, USA).

### 2.3. Statistical Analysis

First, to characterize the effects that walking with the different insole conditions had on the pKAM, iKAM, pKFM, pKEA, pAEM, and pHAM, Cohen’s d effect sizes (ES) were calculated for each patient and gait variable between the 6 combinations of insole conditions (none vs. arch support, none vs. lateral wedge plus arch support, none vs. custom-made, arch support vs. lateral wedge plus arch support, arch support vs. custom, and lateral wedge plus arch support vs. custom). Then, separately for each variable, the distribution of the 60 ES (6 insole combinations × 10 patients) was analyzed with a 4-bin histogram, classifying ES as very small (|ES| < 0.2), small (0.2 ≥ |ES| < 0.5), medium (0.5 ≥ |ES| < 0.8), and large (|ES| ≥ 0.8) [31]. In addition, to help interpret the importance of the changes among insole conditions, the average magnitude of the changes was calculated for each bin.

Second, to describe the individual relationships between the pKAM and the other gait variables, both Pearson correlations and bivariate linear regressions were separately performed for each patient and gait variable. To facilitate the interpretation of the results, the regression coefficients were used to estimate the changes in iKAM, pKFM, pKEA, pHAM, and pAEM occurring along with a 10% reduction in pKAM, the most common target in quantitative pKAM interventions for medial knee OA [32,33].

Third, to characterize the effects of adding a lateral wedge insole on walking biomechanics, the changes in gait variables between the arch support insole and the lateral wedge plus arch support insole were calculated for each patient and variable. Next, for each variable, the effect size of the changes for the 10 patients were quantified using Cohen’s d, and paired Student’s *t*-tests were used to determine if the changes differed from zero. Additionally, the inter-patient relationships between the changes in pKAM and the changes in the other variables were separately assessed with Pearson correlations and bivariate linear regressions for each gait variable.

The normal distribution of the data was confirmed by Kolmogorov–Smirnov tests before using parametric statistics. Gait variables were adjusted for intra-patient variations in walking speed among trials, therefore providing speed-independent figures. Statistical analyses were conducted using Matlab (Mathworks, Natick, MA, USA). The significance level was set *a priori* at 5%. No correction for multiple comparisons was applied, since this study was exploratory, meaning that it aimed at providing insights and not testing specific hypotheses.

## 3. Results

**Figure 1:**
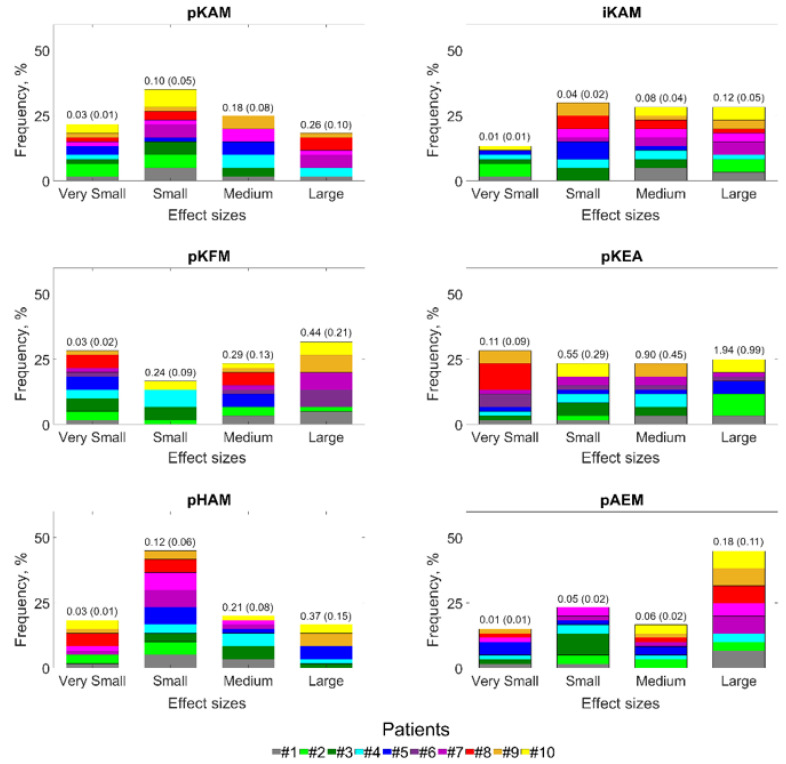
Effect sizes (Cohen’s d) of the intra-patient changes in gait variables between insole conditions. This figure presents the effect sizes distribution using 4-bin histograms, classifying effect sizes (ES) as very small (|ES| < 0.2), small (0.2 ≥ |ES| < 0.5), medium (0.5 ≥ |ES| < 0.8), or large (|ES| ≥ 0.8) [31]. The numbers on top of the bars indicate the average (standard deviation) magnitude of the changes included in the bin. The magnitude of changes is reported in the unit of the gait variables (%BW*Ht, %BW*Ht*s or °).

Walking with different insoles had noticeable effects, with high heterogeneity among patients, on the pKAM as well as on the five other gait variables (Figure 1). For all variables, at least 16.67% of the changes were of large effect size, and at least 16.67% were of medium effect size (at least 36.67% of the changes were of medium-to-large effect size). The mean (± one standard deviation) magnitude of the large effect-size changes in pKAM was 0.26 (0.10) %BW*Ht, which corresponded to changes of 13.25 (5.52)%. The magnitude of the medium effect-size changes, for their part, corresponded to pKAM changes of 7.03 (3.27)%. The magnitudes of the large and medium effect-size changes corresponded to changes of 20.65 (10.55)% and 10.37 (5.22)% for the iKAM, 20.45 (10.93)% and 14.64 (6.31)% for the pKFM, 8.28 (3.63)% and 4.67 (1.89)% for the pHAM, and 71.53 (38.49)% and 32.19 (11.78)% for the pAEM, respectively. On average, the changes of large effect sizes in pKEA had a magnitude of 1.94 (0.99)°, and the changes of medium effect size had a magnitude of 0.90 (0.45)°.

**Figure 2:**
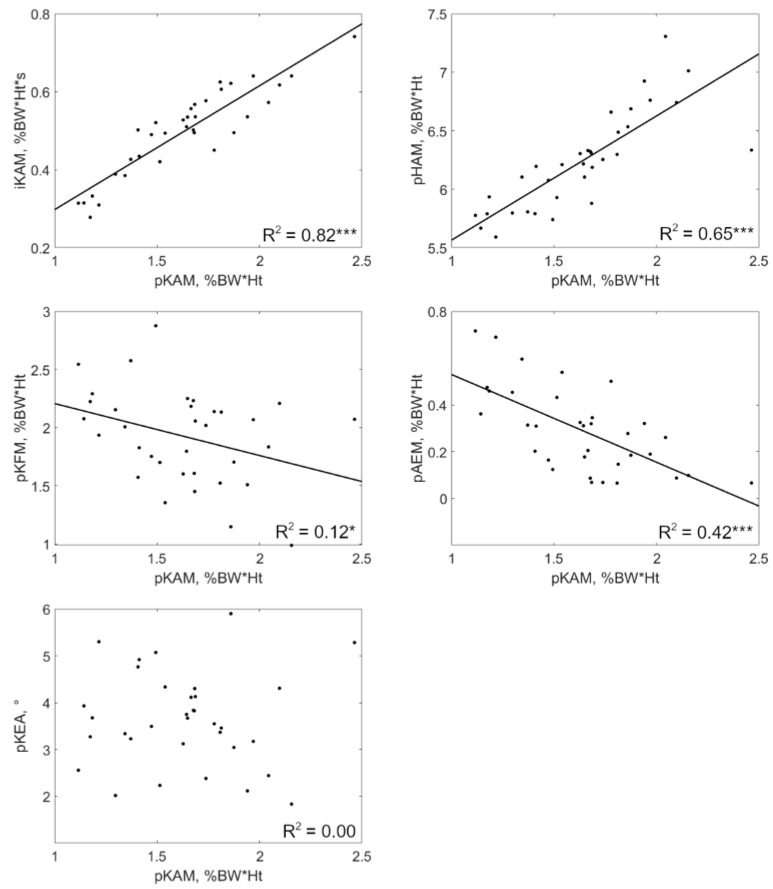
Intra-patient relationships between changes in pKAM and changes in iKAM, pKFM, pKEA, pHAM, and pAEM for a typical patient (#9). Each dot corresponds to a walking trial and the lines to the linear regressions. * *p* ≤ 0.05, *** *p* ≤ 0.001.

Changes in iKAM were statistically significantly positively correlated with pKAM changes in all patients (0.20 ≤ R^2^ ≤ 0.82, *p* ≤ 0.02) (Table 2). Regression coefficients varied in a ratio of 1-to-3 among patients. Indeed, a decrease of 10% in pKAM accompanied iKAM reductions of 4.93% to 15.61%, depending on the patient. Half of the patients reported a statistically significant correlation between changes in pKFM and changes in pKAM (0.11 ≤ R^2^ ≤ 0.60, *p* ≤ 0.05). The correlations were positive for three of them and negative for the two others. For these patients, the regression coefficients indicated that a decrease of 10% in pKAM came along pKFM changes between −18.15% and 108.12%. Changes in pKEA and pKAM were statistically significantly correlated in only one patient (R^2^ = 0.37, *p* < 0.001). This relationship was positive, with a decrease of 10% in pKAM accompanying a 1.71° reduction in pKEA. Changes in pHAM were statistically significantly positively correlated with pKAM changes in all patients (0.17 ≤ R^2^ ≤ 0.87, *p* ≤ 0.03). Depending on the person, a decrease of 10% in pKAM accompanied pHAM reductions between 1.99% and 9.57%. A statistically significant positive correlation between pAEM and pKAM was observed for six of the 10 patients (0.22 ≤ R^2^ ≤ 0.45, *p* ≤ 0.01). For these patients, the regression coefficients indicated that a decrease of 10% in pKAM accompanied pAEM reductions of 13.86% to 95.28%. The relationships between the changes in pKAM and the changes in the other gait variables are graphically presented for a typical patient in Figure 2.

**Figure 3:**
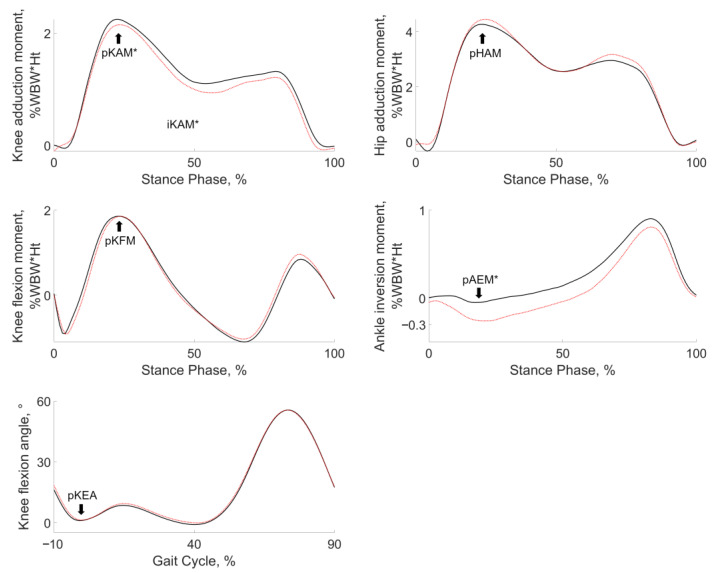
Averaged gait data of all patients walking with an arch support insole (continuous black lines) and with a lateral wedge plus an arch support insole (dotted red lines). Stars (*) indicate statistically significant changes in gait variables with the addition of a lateral wedge (*p* < 0.05; see Table 3 for actual numbers).

Adding a lateral wedge to the arch support insole resulted in statistically significant decreases in pKAM, iKAM, and pAEM for the group of 10 patients (Cohen’s d effect sizes ≤ −0.70, *p* ≤ 0.05) (Figure 3; Table 3). There was a statistically significant inter-patient correlation between changes in pKAM and changes in iKAM (R^2^ = 0.62, *p* = 0.01). This relationship was positive, meaning that patients with the greatest reduction in pKAM were also those with the greatest reduction in iKAM. The regression coefficient for this relationship was 0.47 s, corresponding to 11.03% of iKAM reduction per 10% of pKAM decrease.

## 4. Discussion

By showing that the insole condition had an impact of comparable effect size on the six gait variables, with large inter-patient variability, this study highlights the need to consider biomechanical changes globally, and not only the changes in pKAM. Furthermore, this study also provides insight into the impossibility of limiting measurements to the pKAM and predicting the changes in the other variables based on changes in pKAM. The results were clear in this respect as well. On one hand, there was an absence of or inconsistent intra-patient associations between the changes in pKAM and the changes in pKFM and pKEA, indicating that the changes in pKFM and pKEA cannot be derived from the changes in pKAM, even at the individual level. On the other hand, although the changes in iKAM and pHAM were positively associated with the changes in pKAM for all the patients, the large variations in the association strength among patients corroborated the necessity of measuring all the variables rather than predicting the changes in some variables based on the pKAM. This recommendation is particularly supported by the fact that the methods currently used to measure the pKAM could easily measure other lower-extremity kinetic or kinematic variables without additional burden [34]. Altogether, the observations in this study set an important background for future research by suggesting that larger sets of gait variables should be acquired and analyzed when assessing or designing insole interventions for medial knee OA.

Beyond the purely biomechanical aspects, the findings above are worth interpreting in terms of the perspective of clinics. Doing so highlights three particular avenues that could improve the treatment of knee OA using insoles in the future. First, they encourage reanalyzing prior studies on footwear interventions for medial knee OA to deepen our understanding of the gait variables other than the pKAM. Indeed, numerous high-quality studies have been conducted in this area and could extend our knowledge at limited cost [11,35]. While the present study focused on the iKAM, pKFM, and pKEA based on relationships with knee OA as reported in the literature [5,14,15], considering additional variables when exploring prior studies could prove useful, because it remains unclear which variables play a more important role in the success of footwear interventions. Similarly, considering alternative variables, such as the total joint moment, which combines the pKAM and pKFM [36], or using machine-learning algorithms [37,38] could help establish procedures to define which intervention to prefer in which situation. Considering the individuality of each patient’s gait rather than following data from groups of patients in the literature is another avenue highlighted by the present study to improve the clinical outcomes of insole interventions. This is particularly well-supported by a recent study reporting better outcomes than usual with lateral wedges due to a prescreening of each participant’s gait [39]. This pioneering work that considered the individuality of the patients could even be extended by personalizing the insoles. For example, gait retraining for knee OA already uses patient-specific modifications [33,40]. A third avenue supported by the present study to improve the clinical outcomes of insole interventions in the treatment of knee OA is to widen the evaluation to take into account the biomechanical changes at the other joints. For instance, the intra-patient relationships that were consistently observed in this study between the changes in pKAM and pHAM suggest that at least the integrity of the hip should be assessed before engaging in a pKAM reduction by using insoles to limit side effects at the hip. Unfortunately, with most of the research so far focusing on the knee, little is known about the implications of footwear interventions for the other joints both in terms of function [11,35] and structure [41]. In summary, the present study suggests opportunities to improve the use of insole interventions in the treatment of medial knee OA by considering the biomechanics individually and more globally.

This study also extended the description of the biomechanical effects of lateral wedges. This is important because lateral wedges have been the driver of our understanding of footwear interventions for medial knee OA. Specifically, the present work showed an absence of inter-patient correlation between the changes in pKAM and the changes in pKFM, pKEA, and pHAM. Furthermore, it confirmed the observations of two prior studies regarding a positive association between pKAM and iKAM changes [17] as well as an absence of association between pKAM and pAEM changes [24]. The present study also substantiated the data in the literature, sometimes from a single study, regarding the average effects of lateral wedges on a group of patients with medial knee OA to decrease the pKAM, iKAM, and pAEM and to lead to inconsistent changes in pKFM, pKEA, and pHAM [9,10,11,18]. This variability in the biomechanical response among patients could explain the inconsistent clinical outcomes reported for this intervention [12,13]. Specifically, the current, underspecified objectives of biomechanical interventions for medial knee OA to reduce the pKAM without increasing the pKFM [20,21,33] were already not systematically achieved with lateral wedges. This observation provides additional support to the necessity to develop the knowledge and the tools to map a patient to an effective pair of insoles, as detailed in the previous paragraphs. The individuality in the biomechanical responses to an insole intervention, which suggests the implementation of personalized management, was also well-illustrated by the loss of correlations between the changes in gait variables when the analyses were performed on an inter-patient rather than intra-patient basis. For example, while all the patients reported the changes in pKAM and pHAM to be correlated, the changes in response to the addition of lateral wedges were not correlated among patients.

This study has some characteristics that should be discussed for a proper interpretation of the results. First, in accordance with the study aims, numerous walking trials were collected for a limited number of patients. The results confirmed that the sample size was sufficient to address the objectives, and the main findings would certainly remain the same with a larger study population. Second, the participants of the present study were continuously recruited and, by chance, this led to a gender-imbalanced study population. The data of the female participant (#5) were not obviously different from those of the male participants and was therefore not separately reported. While this was acceptable with respect to the present study objectives, further research aiming at more precisely characterizing the inter-patient variability should account for gender differences in the analyses. The literature mentions additional factors that could influence the variations in response to a mechanical intervention among knee OA patients, such as the static alignment and motion of the foot and ankle during walking [24,42,43,44] or the disease severity (45–47). Future research with larger sample sizes will be necessary to test these and other factors to improve our understanding of the mechanisms leading to differences among patients. Fourth, further work will also be needed to evaluate the long-term responses to insole interventions. Finally, it is important to note that the primary objectives of the study were not to characterize particular types of insoles. In fact, in this study, the insoles were a medium to modify and analyze walking biomechanics. This specific study design and the fact that the insoles were selected to reflect common use in medial knee OA suggest that the findings should be generalizable to other types of insoles. Nevertheless, it cannot be denied that different numbers could be obtained with different insoles.

## 5. Conclusions

By showing that varying the insole conditions in patients with medial knee OA could globally influence the ambulatory biomechanics, this study indicated that limiting measurement to the pKAM might lead to an important loss of information. Further studies on insole interventions for knee OA are therefore encouraged to consider additional gait variables, including the iKAM, pKFM, and pKEA, that have been associated with the disease. This study also shed light on the variability among patients in their responses to insole interventions, stressing the need for and the potential of personalizing the interventions.

## Figures and Tables

**Table 1:** Characteristics of the 10 participants and their study knee.

Characteristics		Values
Participants	Gender, number of males/females	9/1
	Age, years old	57.20 ± 8.32
	Height, m	1.73 ± 0.94
	Weight, kg	88.90 ± 12.58
	Self-selected normal walking speed, m/s	1.31 ± 0.12
Study knees	Side, number of left/right knees	5/5
	Kellgren–Lawrence grades, range: 0–4	2.14 ± 0.90
	Visual Analogue Scale (VAS), range: 0–10	
	Pain	3.30 ± 2.31
	Symptoms	3.20 ± 2.20
	Knee Injury and Osteoarthritis Outcome Score (KOOS), range 0–100	
	General	60.60 ± 15.53
	Pain	67.50 ± 17.25
	Symptoms	69.60 ± 13.71
	Function, daily living	78.30 ± 15.07
	Function, sports and recreational activities	47.50 ± 23.24
	Quality of life	40.60 ± 19.97

Kolmogorov–Smirnov tests indicated that the continuous values were normally distributed. Consequently, they were reported as mean ± one standard deviation.

**Table 2:** Intra-patient relationships between changes in pKAM and changes in other gait variables when walking with different insoles.

Gait Variable	Patient	R^2^	β ^a^	Absolute Changes with 10% pKAM Decrease ^b^	Relative Changes with 10% pKAM Decrease ^c^
iKAM	#1	0.34 ***	**0.24**	**−0.08**	**−9.78**
	#2	0.50 ***	**0.21**	**−0.05**	**−13.47**
	#3	0.59 ***	**0.35**	**−0.08**	**−9.87**
	#4	0.42 ***	**0.55**	**−0.25**	**−11.41**
	#5	0.55 ***	**0.24**	**−0.05**	**−7.88**
	#6	0.41 ***	**0.22**	**−0.03**	**−9.58**
	#7	0.31 ***	**0.20**	**−0.07**	**−5.07**
	#8	0.73 ***	**0.45**	**−0.10**	**−15.61**
	#9	0.82 ***	**0.32**	**−0.05**	**−10.39**
	#10	0.20 *	**0.19**	**−0.06**	**−4.93**
pKFM	#1	0.09	0.52	n/a	n/a
	#2	0.60 ***	**1.23**	**−0.31**	**−18.15**
	#3	0.38 ***	**1.73**	**−0.42**	**108.12**
	#4	0.02	−0.38	n/a	n/a
	#5	0.25 **	**0.66**	**−0.14**	**−8.38**
	#6	0.11 *	**−0.61**	**0.07**	**2.50**
	#7	0.03	−0.33	n/a	n/a
	#8	0.00	−0.01	n/a	n/a
	#9	0.12 *	**−0.45**	**0.07**	**3.79**
	#10	0.00	0.02	n/a	n/a
pKEA	#1	0.04	−0.35	n/a	n/a
	#2	0.02	−0.57	n/a	n/a
	#3	0.37 ***	**7.06**	**−1.71**	**34.02**
	#4	0.00	0.22	n/a	n/a
	#5	0.02	1.73	n/a	n/a
	#6	0.01	0.60	n/a	n/a
	#7	0.00	−0.07	n/a	n/a
	#8	0.00	0.09	n/a	n/a
	#9	0.00	−0.04	n/a	n/a
	#10	0.01	0.55	n/a	n/a
pHAM	#1	0.87 ***	**0.98**	**−0.31**	**−6.02**
	#2	0.84 ***	**1.40**	**−0.35**	**−7.02**
	#3	0.49 ***	**0.74**	**−0.18**	**−6.43**
	#4	0.42 ***	**0.77**	**−0.35**	**−5.38**
	#5	0.37 ***	**1.12**	**−0.25**	**−6.15**
	#6	0.33 ***	**1.01**	**−0.11**	**−2.15**
	#7	0.74 ***	**1.12**	**−0.39**	**−9.57**
	#8	0.61 ***	**0.99**	**−0.22**	**−4.12**
	#9	0.65 ***	**1.06**	**−0.17**	**−2.77**
	#10	0.17 *	**0.34**	**−0.10**	**−1.99**
pAEM	#1	0.09	0.23	n/a	n/a
	#2	0.00	0.02	n/a	n/a
	#3	0.09	0.15	n/a	n/a
	#4	0.30 ***	**0.15**	**−0.07**	**74.22**
	#5	0.45 ***	**0.22**	**−0.05**	**25.84**
	#6	0.22 **	**0.30**	**−0.03**	**13.86**
	#7	0.32 ***	**0.29**	**−0.10**	**95.28**
	#8	0.36 ***	**0.23**	**−0.05**	**19.11**
	#9	0.42 ***	**0.37**	**−0.06**	**20.92**
	#10	0.01	0.05	n/a	n/a

Statistically significant correlations are in bold (* *p* ≤ 0.05, ** *p* ≤ 0.01, *** *p* ≤ 0.001). ^a^ Unstandardized regression coefficients are in seconds for iKAM, in °/(%BW*Ht) for pKEA, and unitless for the other variables. ^b^ Changes in the gait variables of interest associated with a decrease of 10% in pKAM; estimations based on the regression coefficients. Data are in %BW*Ht*s for iKAM, in degrees for pKEA, and in %BW*Ht for the other variables. ^c^ Same as ^b^, but with the changes expressed in percent of the value of the variables of interest.

**Table 3:** Gait changes observed for the group of 10 patients, with the addition of a lateral wedge.

Gait Variable	Values with the Arch Support Insole		Changes with the Addition of a Lateral Wedge			Inter-Patient Correlations between Changes in pKAM and Changes in the Other Gait Variables
	Mean	SD	Mean	SD	ES	R^2^
pKAM	2.62	0.94	**−0.09**	**0.13**	−0.70 *	n/a
iKAM	0.89	0.57	**−0.06**	**0.08**	−0.75 *	0.62 **
pKFM	2.24	1.49	−0.02	0.37	−0.07	0.00
pKEA	0.80	4.75	0.49	1.31	0.37	0.05
pHAM	4.88	1.17	0.10	0.20	0.51	0.25
pAEM	−0.23	0.20	**−0.13**	**0.07**	−1.76 ***	0.14

Statistically significant changes and correlations are in bold (* *p* ≤ 0.05, ** *p* ≤ 0.01, *** *p* ≤ 0.001). Mean and standard deviation values are in %BW*Ht*s for iKAM, in degrees for pKEA, and in %BW*Ht for the other variables. SD: standard deviation. ES: effect size (Cohen’s d).

## Data Availability

The data are not publicly available due to regulatory provisions.
